# Pharmacodynamic and pharmacokinetic properties of the combined preparation of levothyroxine plus sustained- release liothyronine; a randomized controlled clinical trial

**DOI:** 10.1186/s12902-023-01434-y

**Published:** 2023-08-28

**Authors:** Ladan Mehran, Atieh Amouzegar, Seyed Mohsen Foroutan, Safdar Masoumi, Maryam Tohidi, Hengameh Abdi, Ali Aghaei, Amir Esmaeel Saghafinia, Fereidoun Azizi

**Affiliations:** 1 Endocrine Research Center, Research Institute for Endocrine Sciences, Shahid Beheshti University of Medical Sciences, Tehran, Iran; 2 Department of Pharmaceutics, School of Pharmacy, Shahid Beheshti University of Medical Sciences, Tehran, Iran; 3 Protein Technology Research Center, Shahid Beheshti University of Medical Sciences, Tehran, Iran; 4 Prevention of Metabolic Disorders Research Center, Research Institute for Endocrine Sciences, Shahid Beheshti University of Medical Sciences, Tehran, Iran; 5 Noor Research & Educational Institute (TAVAN), Tehran, Iran

**Keywords:** Hypothyroidism, Clinical trial, Combination therapy levothyroxine, Liothyronine

## Abstract

**Background:**

Understanding pharmacokinetics (PK) and pharmacodynamics (PD) of the sustained-release liothyronine (SR-T3) is of paramount importance to design therapeutic regimens that are able to simulate normal thyroid hormone secretion while avoiding excursions in the T3 serum concentration. Here, we designed a parallel randomized clinical trial to characterize the PK and PD of the combined preparations of LT4 + SR-T3 in hypothyroid patients.

**Methods:**

Radioiodine-treated hypothyroid patients over 20 years of age, who attained euthyroidism with LT4 monotherapy were recruited from the Endocrine Clinic in Tehran. The patients were allocated to two intervention groups of group A: 9 µg SR-T3 plus 68.5 μg LT4 (ratio 1:7.5) and group B: 12 µg SR-T3 plus 60 µg LT4 (ratio 1:5), and a control group with LT4 monotherapy. For PD study, thyroid hormone profile was evaluated at 8 and 12 weeks intervals after intervention. To assess PK properties of SR-T3, T3-Cmax, T3-Tmax and AUC_0 − 24_ were calculated at the last visit.

**Results:**

Serum T4 and FT4 concentrations decreased in the intervention groups after 3 months. No significant difference was observed in serum T3 and FT3 concentrations before and after intervention. Serum T3/T4 ratio increased significantly in the intervention groups after intervention, with the highest increase in group B from 8.6 ± 2.03 at baseline to 12.2 ± 1.6. Comparison of trial groups at follow-up showed no differences in serum TSH, T4, T3 and T3/T4 concentrations among different groups. During 24 h, minimal variation in serum T3 concentration was observed in group B with mean ∆T3 of 15.4 ± 10.5 ng/dl. T3-Tmax, T3-Cmax and AUC_0 − 24_ in the combined sustained-release preparation were 4.38 ± 1.1 h., 101.0 ± 5.7 ng/dl and 2257 ± 110 ng.h/L, respectively which were significantly different from the control group.

**Conclusion:**

Combined treatment with a single dose of SR-T3 plus LT4 is associated with increased serum T3/T4 ratio and minimal excursions in serum T3 concentration during 24 h; however, it was not significantly different from the control group. To incorporate sustained-release T3 in the management of hypothyroidism, a higher ratio of SR-T3 to LT4 than that of the previously recommended by the international organizations is suggested.

**IRCT registration number:**

**IRCT20100922004794N13**. https://www.irct.ir/search/result?query=IRCT20100922004794N13. **Registration date: 08/12/2021**.

**Supplementary Information:**

The online version contains supplementary material available at 10.1186/s12902-023-01434-y.

## Introduction

Levothyroxine monotherapy is considered the standard of care for hypothyroidism by professional organizations, whereby the patients achieve the state of euthyroidism through the normalization of TSH, a reliable proxy of euthyroidism [1]. In euthyroid individuals, 80% of T3 (25 µg) is generated from T4 to T3 conversion in kidney and liver cells and 20% ( 5 µg) is secreted from the thyroid gland. Using levothyroxine (L-T4) alone is expected to be a suitable strategy owing to the peripheral conversion of the pro-hormone T4 into the hormonally active T3 by the deiodinase [2, 3]. Of interest, while this treatment modality is safe, well-tolerated, and extremely effective in normalizing TSH, it may not be sufficient to account for the normal thyroidal secretion of T3 and T4. Approximately 10% lower serum T3 levels in LT4-treated patients compared to euthyroid individuals with similar serum TSH values have been reported previously, leading to the skewed T3:T4 ratio toward an increase of T4, while serum T3 levels are normal to low [4–7]. The clinical significance of these deviations in thyroid hormones in LT4-treated patients is not clear.

Clinically, a sizable minority of patients adequately treated with LT4 complain of residual symptoms [8–10]. Higher T4 and lower T3 serum levels reflect a relative imbalance in the thyroid hormone axis homeostasis may be responsible for the persistence of hypothyroid symptoms in some patients. It is unclear to what extent lower serum T3 levels may contribute to the persistent symptoms in hypothyroid patients; however, in an experimental study on thyroidectomized rats with evident tissue hypothyroidism in peripheral cells, tissue euthyroidism occurred only following normalization of serum T3 [11].

The above-mentioned evidence led to the growing interest in combination therapy with LT4 plus liothyronine(LT3); however, multiple randomized controlled clinical trials addressing objective and subjective clinical outcomes did not provide conclusive results [12, 13]. The non-conclusive results, besides heterogeneity in the study population, design, and dosage, may be attributed to the concerns and challenges regarding available oral T3 preparations stemming from the short half-life of T3 with rapid T3 absorption followed by fast metabolization [14–17]. Despite the lack of definite evidence for the superiority of combination therapy, this strategy remains a common practice in hypothyroid patients who remained dissatisfied with LT4 monotherapy, while the use of LT4 + SR-T3 was suggested [18, 19].

To design clinical trials with a greater likelihood of indicating efficacy, the American, European, and British thyroid associations have recently published a consensus statement to promote the development of future clinical trials [20]. The working group for the consensus statement reached 100% agreement that a sustained-release T3 preparation was the preferred preparation for future combined therapy trials, should become available to address shortcomings in the previous trials. The required sustained-release preparations should be able to restore physiological TSH levels in combination with physiological serum T3 to T4 ratio. Therefore, we developed a SR-T3 preparation and formulated different combinations of LT4 plus SR-T3 and in a randomized clinical trial evaluated the PK and PD of the new formulation to find the best combination of LT4 + SR-T3 for future clinical trials.

## Patients and methods

The study was conducted at the Research Institute for Endocrine Sciences, Shahid Beheshti University of Medical Sciences, Tehran, Iran, from January 1, 2022 to 31 June, 2022. Radioiodine-treated hypothyroid patients over 20 years of age, who attained euthyroid status with LT4 monotherapy and serum TSH concentration of 0.5-5 mU/L were recruited from the Endocrine Clinic and referred to the Research Institute for Endocrine Sciences (RIES). The patients with pregnancy, chronic kidney or liver disease, congestive heart failure, cancer, and those taking methimazole, propylthiouracil (PTU), tamoxifen, estrogen, progesterone, and corticosteroids were excluded. Dorsa Pharmaceutical Company and Tavan Institute formulated sustained-release tablets with 7.5, 9 and 12 µg potency.

A parallel randomized controlled clinical trial was applied. Initially, the patients were allocated to the three intervention and one control groups. Intervention groups received three different combinations of LT4 + LT3 yielding ratios of 1:10 (75 µg LT4 + 7.5 µg SR-T3), 1:7.5(9 µg SR-T3 + 68.5 µg LT4) and 1:5 (12 µg SR-T3 + 60 µg LT4). As during follow-up, serum TSH concentration significantly increased in the patients who received 75 µg LT4 + 7.5 µg SR-T3, this group were excluded from the study. Therefore, trial groups in this survey consisted of group A with a daily intake of 9 µg SR-T3 plus 68.5 µg LT4 (ratio 1:7.5), group B with a daily intake of 12 µg SR-T3 plus 60 µg LT4 (ratio 1:5) and a control group with LT4 monotherapy (group C).

**Fig. 1:**
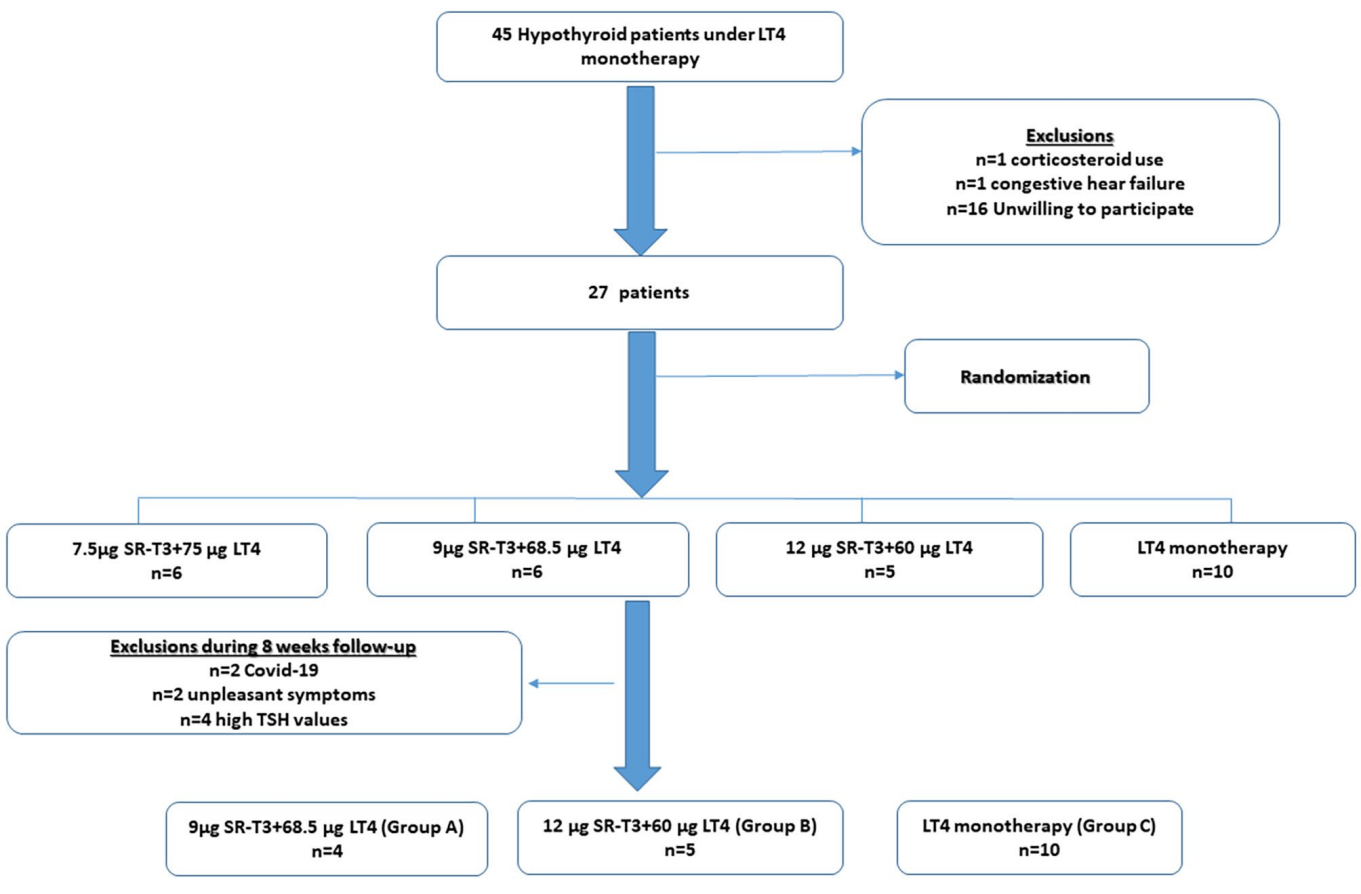
Participants’ flow-diagram

Figure 1 depicts participants flow diagram. Out of 45 hypothyroid patients referred to the clinic, 27 patients with the above-mentioned criteria, who were willing to participate, entered the study of whom 8 patients were omitted during follow-up: two patients due to Covid-19 epidemic conditions, two patients due to unpleasant symptoms and 4 patients due to high TSH values attained during follow-up. Finally, 19 patients were allocated to two intervention (n = 9) and one control group (n = 7). The treatment was allocated based on the pre-specified random allocation. The participants were evaluated for thyroid related signs and symptoms and thyroid hormone biochemical assessments at the baseline and two follow-ups at 8 and 12 weeks after intervention; for adjusting drug dosage, there was a TSH check-up after 6 weeks. To assess the pharmacokinetics of different combined preparations of LT4 + SR-T3, in the last follow-up visit, blood sampling was performed after 12 h fasting; then the patients received the allocated treatment followed by the repeated measurements after 1, 2, 4, 6, 8, and 24 h. One of 5 patients in the intervention group B, and 7 of 10 patients in the control group refused to participate in the PK study.

At the baseline visit, after taking informed written consent, blood pressure, weight, and height were measured. The trained staff filled the questionnaire regarding demographic and thyroid symptoms. Patients were allocated to three intervention groups and one control group using stratified randomization. Two stratifications were made based on gender. Under each sex subgroup, patients were randomly assigned to three treatment groups using the random table allocation.After implementation of randomization and specific coding, the subjects were assigned to two intervention and one control groups using allocation concealment. The standard method of ensuring allocation concealment was sequentially numbered. The staff member not involved in the trial was instructed to keep the list private and only revealed a treatment allocation after receiving information showing that the patient is eligible and has consented to the trial. The patients and the investigators were kept from knowing who was assigned to which treatment. All groups received identical tablets in physical appearance, taste, and smell to fulfill blinding.

The participants were evaluated at baseline and two consequent follow-ups on the 8^th^. and ^12th^. weeks. The patients were visited after 6 weeks to measure serum TSH and assess therapy adherence. To ensure compliance with drug therapy, the responsible person checked the drug package and counted the number of pill intakes by direct questioning in two weeks intervals by phone call and pill counting at the last visit. Drug dosage was adjusted to maintain serum TSH concentration within 0.5-5 mU/L. At baseline and two follow-ups 8 and 12 weeks later, body weight and blood pressure were measured and venous blood samples were collected after a 12-hour fasting. The serum samples maintained in microcentrifuge tubes at -20ºC to measure serum TSH, total T3, total T4, free T4, and free T3. After fasting blood sampling at the last visit, the patients received the allocated treatment at 8 am, and the blood sampling was taken 6 times during a 24 h period at 9 am, 10 am and 12 am, 14–16 pm, and the next day at 8 am, to measure serum TSH, T4, free T4, T3, and free T3 concentrations in all samples to calculate T3/T4 ratio, time for T3 to reach maximum concentration (T3-Tmax), maximum serum concentration of T3 (T3-Cmax) and the area under the plasma concentration time curve over 24 h (AUC_0 − 24_).

### Outcome measures

Primary outcome was set to find the best LT4 + SRT3 combination to maintain normal serum concentration of TSH, T4, T3, free T4, free T3 and T3/T4 ratio. Secondary outcome was to find the best LT4 + SRT3 combination which provide most favorable T3- Cmax, T3-Tmax, and AUC and patient preferences. The study design was registered by the Iranian Registry of Clinical Trials, IRCT20100922004794N13 (dated: 08/12/2021) available at: “https://www.irct.ir/search/result?query=IRCT20100922004794N13”. This study was conducted in concordance with the Helsinki Declaration ethical principles, and all procedures on the study participants were approved by the National Research Council of the Islamic Republic of Iran (IR.SBMU.ENDOCRINE.REC.1400.031), the Human Research Review Committee of the Endocrine Research Center, Shahid Beheshti University, Tehran, Iran. Trial participants signed informed consent forms at baseline, and their personal information would remain strictly confidential.

### Drug formulation and dosing

The formulation of liothyronine sodium sustained-release tablets was developed by Noor Research & Educational Institute (TAVAN). Using Avicel PH-102 and HPMC in formulation of tablets sustained the drug release of T3 for over 10 h. The in vitro dissolution study was carried out in 500 ml, pH 6.8 phosphate buffer using United States Pharmacopeia Apparatus I, basket method, at a speed of 100 rpm and temperature of 37 C. Levothyroxine sodium (LT4) plus SR-T3 was administered based on the previous levothyroxine dosage which was at a weight-based dose of ~ 1.6 mcg/kg. Each combination was equal to 100 µg Levothyroxine.

The 2012 ETA, suggested L-T4 + L-T3 combination treatment with a dose ratio between 13:1 and 20:1 by weight [21], however in the first study by Hennamenn et al., combination therapy with SR-T3 + LT4 with a ratio of 1:20 [22], mean T3 to T4 ratio did not reach to mean values in normal subjects. Therefore, we examined the different combinations with higher SR-T3 ratios to see whether more appropriate serum T3 to T4 ratio would be achieved. In our study, examining combined therapy with SR-T3 + LT4 with ratios of 1:14 and 1:10 could not attain objectives, therefore, two combinations of 1:~8 and 1:5 ratios for SR-T3 and LT4, respectively, were chosen for this study.

### Laboratory measurements

All laboratory measurements were determined on -20ºC stored serum samples. The thyroid function tests including TSH, T3, T4, and free hormones were measured by electrochemiluminescence immunoassay method using Roche Diagnostics kits on the Cobas e- 411 automated analyzer (Roche Diagnostics, GmbH, Mannheim, Germany). The sensitivity of TSH, T3, T4, FT3, and FT4 assays were 0.005 µIU/mL, 0.195 ng/mL, 0.420 µg/dL, 0.6 pmol/L, and 0.5 pmol/L, respectively. Lyophilized quality control materials (Lyphochek Immunoassay Plus Control, Bio-Rad Laboratories) in three different concentrations were used to monitor accuracy of measurements. The intra- and inter-assay coefficients of variation (CVs) were respectively 2.8% and 3.1%.

for TSH, 2.3% and 3.0% for T3, 1.7% and 2.6% for T4, 2.1% and 3.9% for FT3, and 1.0% and 2.9% for FT4. Mean values and the reference ranges of thyroid hormones obtained from the Tehran Thyroid Study (TTS) were TSH (1.77 ± 1.24; 0.32–5.06 mU/L) and FT4 (1.19 ± 0.16; 0.91–1.55 ng/dl) [23]. Normal values (mean ± SD) of T3, T4 and T3/T4 ratio in 73 normal euthyroid subjects in the previous study were 131 ± 38 ng/dL, 8.96 ± 3.00 µg/dL and 14.62 ± 5.17 ng/µg, respectively.

### Statistical analysis

All analyzes were performed using STATA software version 14. Quantitative variables were summarized using mean, standard deviation and median. Qualitative variables were reported in frequency and percentage. Baseline characteristics between the three groups were tested using ANOVA. The comparison within groups was performed by two-tailed paired Student’s t-test or with the Wilcoxon rank t test. Chi-square person test were used to compare the frequencies between classification variables. The difference between the three groups was tested using the ANOVA test and Benferoni post hoc test. P value less than 0.05 was considered statistically significant. The maximum observed concentration (C-max) and the time at which this occurred (T-max) were calculated. The area under the plasma concentration–time curves from zero to the last sampling time AUC0-24 was calculated according to the trapezoidal rule using the actual times of measurements. Analysis of covariance (ANCOVA) was performed on AUC, Tmax and Cmax using STATA software version 14. ANOVA results revealed the effect of the sources of variation like sequence, subjects in a sequence, period and formulation on the bioequivalence data. The results of ANCOVA were calculated at 5% level of significance.

## Results

Nineteen hypothyroid patients adhered to the allocated therapy until the end of the study. The baseline characteristics of the intervention and control groups were presented in Table 1. The mean age of the study population was 59.1 ± 7.8 y. of whom 13 patients (68.4%) were women. The mean BMI value was 28.2 ± 4.5 kg/m^2^. There was no difference in age, sex, BMI, and serum concentrations of thyroid hormones among trial groups at baseline.

**Table 1:** Baseline characteristics of the intervention and control groups at baseline

	Total n = 19	Group A n = 4	Group B n = 5	Group C^†^ n = 10	p
Age (year)^*^	59.1 ± 7.8	57.5 ± 12.6	62.0 ± 6.3	57.3 ± 7.5	0.7
Women, n(%)	13 (68.4)	2 (50)	5 (100.0)	6 (60.0)	0.4
BMI (kg/m^2^)^*^	28.2 ± 4.5	32.2 ± 5.9	28.4 ± 2.4	26.2 ± 3.82	0.6
TSH (mU/L)^*^	1.36 ± 1.34	0.84 ± 1.08	0.82 ± 0.72	1.84 ± 1.56	0.3
Total T4 (µg/dL)^*^	9.35 ± 2.12	10.03 ± 2.31	10.19 ± 1.05	8.66 ± 3.37	0.3
Total T3 (ng/dL)^*^	102.71 ± 24.51	95.30 ± 14.15	87.68 ± 21.84	113.19 ± 25.51	0.1
Free T4 (pmol/L)^*^	18.21 ± 4.50	20.82 ± 3.93	21.0 ± 1.34	15.75 ± 4.50	0.03
Free T3 (pmol/L)^*^	4.21 ± 0.98	4.17 ± 0.69	4.22 ± 1.50	4.26 ± 0.31	0.9
T3/T4 ratio(ng/µg)^*^	11.60 ± 4.49	10.04 ± 3.44	8.62 ± 2.03	13.71 ± 4.85	0.6
FT3/FT4 ratio^*^	0.41 ± 0.10	0.44 ± 0.15	0.41 ± 0.12	0.42 ± 0.09	0.7

^*^values are based onmean ± SD; Group A (9 µg SR-T3 + 68.5 µg LT4), Group B (12 µg SR-T3 + 60 µg LT4), Group C (LT4 monotherapy)

Two combined preparations of LT4 + SR-T3 did not have enough potency to attain normal TSH values; however, after increasing the dosage, serum TSH concentrations reached values within the normal ranges. Table 2 compares serum TSH, TT3, TT4, FT3, and FT4 concentrations and T3/T4 ratios between the intervention (A, B and A + B) and control groups at the baseline and follow-ups before and after 12 weeks of intervention. Non-significant increase was observed in serum TSH values in group A (from 0.8 ± 1.08 to 5.4 ± 4.7 mU/L), and B (from 0.8 ± 0.7 to 3.2 ± 1.5 mU/L). After intervention, serum free and total T4 concentrations decreased in the intervention groups (A&B) with a significant reduction only in group B (10.1 ± 1.05 to 7.5 ± 0.3 µg/dL). Serum T3 concentration increased only in group B after intervention, however, the before-after difference was not significant. Serum T3/T4 ratio increased significantly in both groups A and B after intervention, with the highest increase in group B (from 8.6 ± 2.03 to 12.2 ± 1.6 ng/µg). Although there was significant decrease in serum T4 values and increase in T3\T4 ratio within the intervention groups, no differences were observed in serum concentrations of TSH, T4, T3 and T3/T4 ratio among different groups at follow-up (Supplement 1).

**Table 2:** Serum concentrations of TSH, Total T4, total T3, free T4, free T3, FT3/FT4 and T4/T3 at the first and last follow-up in intervention and control groups

	Group A *(9 µg SR-T3 + 68.5 µg LT4)*	Group B (12 µg SR-T3 + 60 µg LT4)	Group A + B *Combination therapy*	Group C *(LT4 monotherapy)*
	Mean ± SD	Mean ± SD	Mean ± SD	Mean ± SD
TSH (mU/L)				
Baseline	0.84 ± 1.08	0.82 ± 0.72	0.83 ± 0.84	1.84 ± 1.56
12 weeks	5.40 ± 4.73	3.26 ± 1.55	4.20 ± 3.29^*^	2.22 ± 1.16
T4(µg/dL)				
Baseline	10.03 ± 2.31	10.19 ± 1.05	10.12 ± 1.59	8.71 ± 2.28
12 weeks	7.43 ± 1.41	7.56 ± 0.35^**^	7.50 ± 0.89^***^	7.78 ± 2.13
T3(ng/dL)				
Baseline	95.30 ± 14.15	87.68 ± 21.84	91.06 ± 18.1	112.29 ± 23.4
12 weeks	87.45 ± 9.15	92.68 ± 12.16	90.35 ± 10.6	109.28 ± 22.3
T3/T4(ng/µg)				
Baseline	10.04 ± 3.44	8.62 ± 2.03	9.29 ± 2.65	13.71 ± 4.85
12 weeks	12.03 ± 2.31^*^	12.26 ± 1.63^*^	12.15 ± 1.82^**^	12.0 ± 1.76
FT4(pmol/L)				
Baseline	20.82 ± 3.93	21.0 ± 1.34	20.95 ± 2.58	15.75 ± 4.51
12 weeks	18.14 ± 4.99	15.57 ± 0.94^**^	16.71 ± 3.41^*^	17.24 ± 1.30
FT3(pmol/L)				
Baseline	4.17 ± 0.69	4.22 ± 1.50	4.19 ± 1.14	4.26 ± 0.31
12 weeks	3.76 ± 0.67	3.89 ± 0.58	3.82 ± 0.58	4.21 ± 0.08
FT3/FT4				
Baseline	0.44 ± 0.15	0.41 ± 0.12	0.42 ± 0.12	0.42 ± 0.09
12 weeks	0.21 ± 0.02	0.25 ± 0.04^*^	0.23 ± 0.03^**^	0.25 ± 0.02

Group A: 9 µg SR-T3 + 68.5 µg LT4, Group B: 12 µg SR-T3 + 60 µg LT4; Group C: LT4 monotherapy; data in intervention groups A and B are pulled (Group A + B).Analysis based on Wilcoxon rank t test, * p-value < 0.05 ** p-value < 0.01 *** p-value < 0.001

**Fig. 2:**
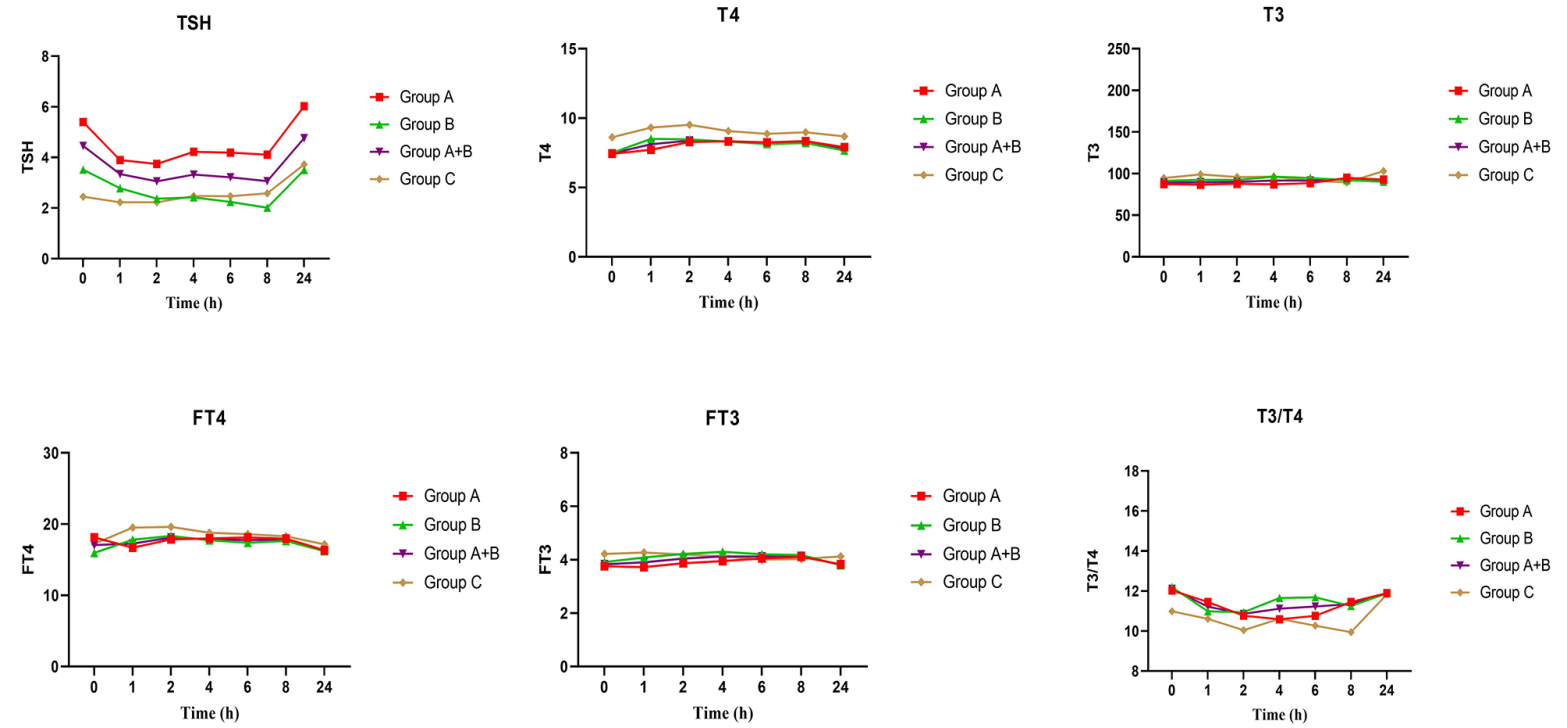
Serum concentrations of TSH, Total T4, total T3 and T3/T4 ratio in intervention groups of A,B, A + B and control groups at last follow-up day; baseline and after 1, 2, 4, 6, 8 and 24 h. Group A: 9 µg SR-T3 + 68.5 µg LT4; Group B: 12 µg SR-T3 + 60 µg LT4; Group C: Control; Group A + B included the patients in both groups A&B

The results of the serum concentrations of TSH, total T4, total T3, and T3/T4 ratio in the intervention and control groups in serial measurements for the PK study at last follow-up are presented in Fig. 2. AUC_0 − 24_, T-max, and C-max for T3, T4 and T3/T4 ratio in the intervention and control groups are presented in Table 3. T3-Tmax in intervention and control groups were 4.4 and 16 h, respectively. T-max for T3/T4 ratio was achieved after 10.5 h in the intervention group (A + B) and after 16 h in the control group and. Mean profile change (Minimum, Maximum) of serum T3 concentration during 24 h in the groups A, B and C were 5.28 (3.71, 8.12), 6.85 (5.34, 9.10) and 9.77 (7.21, 12.17), respectively. Mean ∆T3(maximum - minimum) in group A, B and C were 15.87 (± 5.93), 15.48 (10.59) and 20(3.20) ng/dL, respectively.

**Table 3:** Area under curve, T-max and C-max for T3, T4 and T3/T4 ratio in intervention (group A and B) and control (group C) groups

Pharmacokinetic T3	Group A	Group B	Group A + B	Group C	P-value
AUC_0 − 24_	2346.5 ± 212.3	2168.8 ± 81.5	2257.7 ± 110	2236.8 ± 76.3	0.7
T- max (h)	5.5 ± 1.8	3.2 ± 1.1	4.38 ± 1.1	16.0 ± 8.0	0.12
C-max (ng/dL)	97.8 ± 8.9	104.2 ± 8.2	101.0 ± 5.7	105.7 ± 4.7	0.8
Pharmacokinetic T4					
AUC_0 − 24_	198.6 ± 14.1	196.6 ± 9.6	197.6 ± 7.9	217.3 ± 6.6	0.4
T-max (h)	5.5 ± 0.9	3.2 ± 1.6	4.3 ± 0.9	2.0 ± 0.0	0.2
C-max(µg/dL)	8.5 ± 0.6	8.8 ± 0.3	9.5 ± 0.3	9.5 ± 0.3	0.4
Pharmacokinetic T3/T4					
AUC_0 − 24_	288.1 ± 31.6	266.0 ± 15.9	277.0 ± 16.9	247.3 ± 1.2	0.5
T-max (h)	7.5 ± 5.7	13.5 ± 6.2	10.5 ± 4.0	16.0 ± 8.0	0.7
C-max(ng/µg)	12.3 ± 1.2	12.7 ± 1.1	12.5 ± 0.7	12.1 ± 0.1	0.9

Group A: 9 µg SR-T3 + 68.5 µg LT4, Group B: 12 µg SR-T3 + 60 µg LT4; Group C: LT4 monotherapy; the values are expressed as mean ± sd; data in intervention groups A and B are pulled (Group A + B)

At the end of the study, among 9 patients who underwent combined therapy, three preferred LT4 monotherapy, one preferred combination therapy and five patients reported no preference.

## Discussion

Decreased serum T4 concentrations and increased serum T3 concentration in treatment with 12 µg SR-T3 plus 60 µg LT4 led to a significant increase in serum T3/T4 ratio after intervention; however, serum T3/T4 ratio was lower than the normal serum T3/T4 ratio in euthyroid subjects (~ 1:14–15) and no difference was observed in serum T3, T4 and T3/T4 ratio after intervention between CBT (combination therapy) and LT4 monotherapy groups. Single dose administration of LT4 + SR-T3 was associated with more improved T3-derived Tmax(~ 4–5 h) and Cmax (~ 105 ng/dl) while serum T3 concentration remained within normal ranges with no fluctuation for 24 h after achieving maximum level.

The only study by Hennemann et al. with randomized crossover design on fifteen patients (mean age of 50 y.) with primary hypothyroidism showed that T4 plus sustained-release T3 (1:20 ratio) improved serum TSH, T4, and T3 concentrations and the T4/T3 ratio compared to treatment with LT4 alone, while non-physiologic T3 peaks were avoided [24]. In the current survey, despite a significant increase in serum T3/T4 ratio in intervention groups, no difference was observed in serum thyroid hormone profile and T3/T4 ratio between CBT and LT4 monotherapy groups at follow-up, however similar to the Hennemann study sustained-release T3 did not indicate serum T3 peaks during 24 h. Our study had the preference of using more appropriate SR-T3 dosage and including only patients in whom thyroid gland was ablated by radioactive iodine; while in the study by Hennemann, hypothyroid patients with heterogeneous etiology, e.g. Hashimoto thyroiditis, were included in whom endogenous thyroid hormone might interfere with the results. In both study despite normal serum TSH concentration achieved, mean serum T3/T4 ratio did not reach mean values of euthyroid subjects. To address the issue, either we should increase the ratio of LT4 or slightly increase SR-T3. Increase LT4 values besides reducing the ratio of T3/T4, may inactivate type 2 deiodinase and reduce active T3 in peripheral tissues [11]. Therefore, it seems that combination therapy with higher T3/T4 ratio might be beneficial.

Although serum TSH values are comparable and within normal ranges in LT4-treated patients, LT4 as an exogenous pro-drug cannot simulate serum thyroid hormone profile and circadian rhythm in the native euthyroid state.Serum TSH values in the previous clinical trials varied in patients under LT4 + LT3 combination therapy [25]. In this study, serum TSH levels in the intervention group increased to abnormal levels and after adjusting the dosage restored to normal values, which showed insufficient potency in the combined tablets. As was expected and shown in the literature, we found a decline in serum T4/FT4 values in LT4 + SR-T3 treated patients because of lowering LT4 dosages administered to these patients [25]. We found an increase in serum T3 in those who received CBT with 1:5 ratio (group B) in line with the results of about half of the previous clinical trials involving LT4 + LT3 combination therapy indicating an increase in serum T3 compared to LT4-treated patients; however, most of these studies used a plain form of LT3 [26–33]. T3 to T4 ratio increased in the intervention group, however, the ratio was similar to the control group and lower than those with the native euthyroid states in line with a few previous reports [3, 28].

The studies on the PK properties of sustained-release liothyronine are more coordinated; however, apart from the current study, there is only one other study which assessed PK of SR-T3 in combined preparation with LT4 [24]. In the current study, PK of combination therapy with sustained-release T3 showed minimal excursions with serum T3 steadily increased and reached the peak level (104 ng/dl) after 4–5 h and remained stable for 24 h (groups A & B). In Henneman’s study combination therapy with sustained-release T3 decreased the T3 peak in the serum (Cmax) by approximately 9% and prolonged the time to Cmax from ~ 3.2 to 5 h by slowing down the release of LT3 in the intestine [24]; Cmax, Tmax, and AUC0-24 for liothyronine in the LT4 + SR-T3 group were 1.67 ± 0.06 nmol/L(~ 46.1 ng/dl), 4.97 ± 0.57 h, and 36.65 ± 1.43 nmol.h/L(~ 1056 ng.h/L), respectively; while the T3-Cmax was comparable to this study, the documented Cmax and AUC were lower due to the lower dose of SR-T3 (LT3 to LT4 ~ 1:20). In another study on 28 thyroidectomized patients given 3,5,3′-triiodothyronine sulfate (T3-S) orally, serum T3 levels, generated from T3-S desulfation, rapidly increased with an early peak of 2–4 h; followed by variable plateaus which sustained for 48 h in circulation; The ratio of T3-S variations, as estimated by the Cmax and AUC_0− 48_, was proportional to the given dosage and the authors suggested that adequate administration of oral T3-S might consider a more suitable treatment for hypothyroidism [34]. However, the sustained-release of T3 was not seen in a study on 43 euthyroid participants who received a single 50 mcg dose of sustained-release liothyronine (Thyromax®; BCT303, made with microcrystalline cellulose and magnesium stearate), but once-daily LT3 dosing resulted in a sustained lowering of TSH and stable regulation of other T3 regulated gene products, even without achieving steadily maintained serum T3 concentration [14].

The current study is strengthened by including hypothyroid patients under LT4 monotherapy due to radioactive iodine intake for treating Graves’ Disease with no or negligible residual secretion of endogenous T3 and T4 and using the modern technique for measurement of thyroid hormones. Evaluation of the two combinations of the LT4 + SR-T3 enabled us to choose a more appropriate combination based on the participant’s body weight and not a fixed dosage such as the previous studies. Also, to achieve equilibrium in thyroid hormones, the PK study was performed after three months of treatment and not by a single-dose drug intake. However, the study is limited by evaluating PK study only within 24 h and the low potency of the LT4 component in the combined therapy which made us to increase the total dosage to restore euthyroidism.

In conclusion, combined treatment with levothyroxine plus sustained-release liothyronine in hypothyroid patients with ratios of 1:5 might restore euthyroidism in hypothyroid patients yielding more improved T3/T4 serum ratio and T3-derived pharmakikinetics,while keeping serum T3 concentrations relatively stable within 24 h with minimal excursions. Single dose administration of LT4 + SR-T3 maintains serum T3 concentration approximate to normal ranges up to 24 h with minimal variation in serum T3 not exceeding 25 ng/dl indicates longer duration of the SR-T3 release in the intestine compared to other preparations. Although maximum serum T3 concentration (Tmax) achieves during 3–4 h, there is minimal difference in serum T3 concentrations at serial measurements during 24 h. Significant increase in serum T3/T4 ratio in combined therapy which is still lower than mean values in euthyroid individuals, could be translated into the assumption that administration of higher ratios of SR-T3 in combination therapy should be provided to achieve the physiological ratio of T3/T4 in treated hypothyroid patients. After attainment of the best appropriate preparation, designing and conducting appropriate randomized controlled clinical trials to compare the efficacy outcomes of combination therapy with sustained- release liothyronine are highly warranted to ensure sustainable delivery of T4 and T3 to thyroid hormone target issues.

### Electronic supplementary material

Below is the link to the electronic supplementary material.

Supplementary Material 1

## Data Availability

Some or all datasets generated during and/or analyzed during the current study are not publicly available but are available from the corresponding author on a reasonable request.
