# Supplementary figures and images for: Pharmacodynamic and pharmacokinetic properties of the combined preparation of levothyroxine plus sustained- release liothyronine; a randomized controlled clinical trial
Source: BMC Endocr Disord. 2023 Aug 28;23:182. doi: 10.1186/s12902-023-01434-y (PMC10463362; doi:10.1186/s12902-023-01434-y)

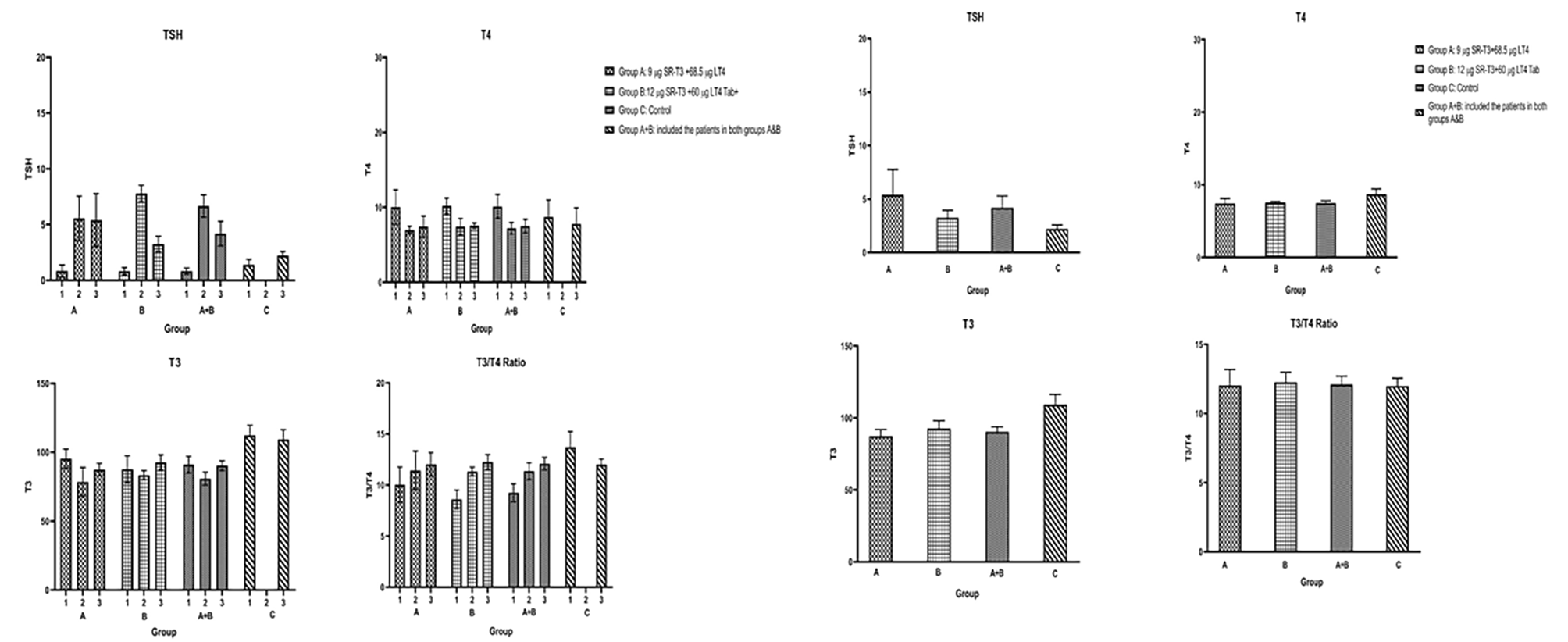

Supplement: Supplementary file 1 — Supplementary Material 1 [file 12902_2023_1434_MOESM1_ESM.png]
